# Complete genome sequence of Shewanella algae strain 2NE11, a decolorizing bacterium isolated from industrial effluent in Peru

**DOI:** 10.1016/j.btre.2022.e00704

**Published:** 2022-01-31

**Authors:** Wendy C. Lizárraga, Carlo G. Mormontoy, Hedersson Calla, Maria Castañeda, Mario Taira, Ruth Garcia, Claudia Marín, Michel Abanto, Pablo Ramirez

**Affiliations:** a Laboratory of Molecular Microbiology and Biotechnology, Faculty of Biological Sciences, Universidad Nacional Mayor de San Marcos, Lima, Perú.; b Núcleo Científico y Tecnológico en Biorecursos - BIOREN, Universidad de La Frontera, Temuco, Chile.

**Keywords:** Shewanella algae, whole-genome sequencing, dyes, decolorization

## Abstract

Shewanella is a microbial group with high potential to be applied in textile effluents bioremediation due to its ability to use a wide variety of substrates as a final electron acceptor in respiration. The present research aimed to describe a new strain, Shewanella algae 2NE11, a decolorizing bacterium isolated from industrial effluent in Peru.

S. algae 2NE11 showed an optimal growth under pH 6-9, temperature between 30-40 °C, and 0-4 % NaCl. It can tolerate high concentrations of NaCl until 10% and low temperatures as 4 °C. It decolorizes azo and anthraquinone dyes with a decolorization rate of 89-97%. We performed next-generation sequencing (Pacific Bioscience®) and achieved its complete genome sequence with a length of 5,030,813bp and a GC content of 52.98%. Genomic characterization revealed the presence of protein-coding genes related to decolorization like azoreductase, dyp-peroxidase, oxidoreductases, and the complete Mtr respiratory pathway. Likewise, we identified other properties such as the presence of metal resistant genes, and genes related to lactate and N-acetylglucosamine metabolism. These results highlight its potential to be applied in the bioremediation of textile effluents and guide future research on decolorization metabolic pathways.

## Highlights

• The complete genome sequence of Shewanella algae strain 2NE11 was recovered and characterized.
• Phenotypic characterization including growth conditions and decolorization rate under various types of dyes were evaluated.
• A variety of genes associated with decolorization, metal resistance, carbohydrate metabolism and CRISPR-Cas system were identified.
• Two genomic islands were identified harboring genes related to metabolic processes and horizontal gene transfer suggesting their role of the strain in environmental adaptations.
• Due to its phenotypic and genomic features, S. algae 2NE11 could be used in efficient biotechnological bioremediation operations.

## 1 Introduction

There are 100,000 different dyes worldwide, mainly used in the textile, plastic, food, and cosmetic industry; among them, azo and anthraquinone dyes are considered the most important, long, diverse, and recalcitrant xenobiotic group mainly used by the textile industry for their high efficiency and low cost [9, 13, 31, 36, 45]. Previous research estimated that approximately 2% of basic dyes and 50% of reactive dyes get lost during the textile industry staining process, percentage that represent a big social concern in contamination [36]. Some dyes are considered harmful to aquatic and human organisms due to their toxicity, mutagenicity, and carcinogenesis [11, 36, 43].

*Shewanella* genus has over 60 species; they are gram-negative bacilli with a size of 1-3 µm and mobile by a polar flagellum, with a facultative anaerobic metabolism; and they are found in a great diversity of habitats such as sand, water, and marine sediments, coal mines, oil, among others [57]. The genus has a high potential for bioremediation due to its genomic versatility and the capacity for dissimilatory metabolism of a wide diversity of compounds such as toxic elements and insoluble metals [14, 18, 25].

Shewanella has been reported as a potential bioremediation agent for azo and anthraquinone dyes under high salinity, microaerophilic, aerobic, and anaerobic conditions [20, 27, 56]. The decolorization process in bacteria involves dye reduction by enzymes such as azoreductases, laccases, and peroxidases. Some enzymes, such as manganese peroxidase (MnP), polyphenol oxidase (PPO), tyrosinase, veratryl alcohol oxidase, and lignin peroxidase (LiP), were also associated with decolorization in other microorganisms [30, 42]. Although the biodegradation mechanisms of azo dye by Shewanella species have been studied, genomic characterization focused on identifying the enzymes involved in decolorization of azo dyes by Shewanella strains remains little explored.

This work aims to describe *Shewanella algae* 2NE11 genomic features and highlight its potential application in decolorization process.

## 2 Materials and Methods

### 2.1 Sampling and Isolation

We isolated *S. algae* 2NE11 from an olive processing company effluent in the city of La Yarada - Los Palos (Tacna - Peru) (18°12’40.1′' S 70°30’33.3). Some physicochemical parameters as DBO and DQO (mg/L) were determinate during sample collection. The sample was grown in minimal medium broth (g/L: KH_2_PO_4_:1; CaNO_3_:0.03; MgSO_4_: 0.2; K_2_HPO_4_:2.77; NH_3_SO_4_:1) for 48 h at 25°C. Subsequently, bacterial culture was grown in nutritive agar for 48h. Then, colonies were separated based on their morphology and were examined for potential to decolorize dyes efficiency. Finally, we selected strain 2NE11 based on its high decolorization efficiency for further investigation.

### 2.2 Growth conditions and biochemistry assays

We evaluated the following parameters: temperature (4, 30, 37, 40°C), pH (5, 6, 7, 8, 9, 10, 11) and NaCl concentration (0, 1, 2, 3, 4, 5, 6, 7, 8, 10 %) after 24 h of incubation to determine the optimal growth condition for strain 2NE11 in Luria Bertani broth (LB) modified [39, 50]. The principal biochemical tests were performed as described previously [4, 24]. Besides, we determined the carbohydrates utilization profile in the Hugh Leifson culture medium supplemented with 0.1% carbohydrate (% w/v). The carbohydrates evaluated were: D-galactose, D-fructose, D-glucose, maltose, D-mannitol, N-acetyl glucosamine, sucrose, and DL-lactate [24]. A biological triplicate assay and a control group was included in all the experiments.

### 2.3 Decolorization kinetics

Decolorization kinetics were performed in 100 mL of ZZ broth pH 7.0 (g/L: (NH_4_)2SO_4_: 0.5; KH_2_PO_4_: 2.66; Na_2_HPO_4_.2H_2_O: 4.32; yeast extract: 5) supplemented with 100 mg/L of dye and inoculated with 5% bacterial culture harvested at mid-log phase (% v/v) [60]. Each flask was homogenized and dispensed in tubes with 5 mL and incubated in static conditions at 30°C during 12 h. After centrifugation at 10°C, 7850 rpm for 10 minutes, the absorbance was obtained at a maximum wavelength of each dye evaluated (Direct Blue 71: 574nm; Methyl Orange: 470nm; Bright Blue Remazol: 608nm; Yellow Procion HEXL: 422nm). The assay was performed by triplicate and a negative control group (without bacterial inoculation) [46, 54].

### 2.4 DNA extraction and genome sequencing

The strain 2NE11 was inoculated 5% (v/v) in ZZ broth pH 7.0 supplemented with Direct Blue 71 dye at 100 mg/L and incubated at 30°C for 12h.Then, 5 mL of culture was centrifuged at 10°C and 7850 rpm for 10 minutes. The pellet obtained was used for total DNA extraction following the standard protocol of the PureLink™ Genomic DNA Mini Kit (Invitrogen^TM^). NanoDrop Lite was used to obtain DNA quantity and quality. DNA integrity was evaluated through an agarose gel electrophoresis. The whole-genome library was generated by a 20Kb SMRTbell and sequencing by Single-Molecule Real-Time (SMRT) with RSII and C4-P6, using the Pacific Bioscience® technology at Macrogen (Korea).

### 2.5 Genome assembly and functional annotation

Genome assembly was made with Unicycler using conservative mode and additional polishing steps with Quiver [51]. The program Quast [16] compared the measure of assemblies quality obtained by Unicycler. A genomic map was depicted with BRIG [1].

The pipeline Prokka and the Rapid Annotation Subsystems Technology (RAST) [2, 40] were used to annotate the complete genome sequence of strain 2NE11. Final genome annotation was obtained by the Prokaryotic genome annotation pipeline (PGAP) [47]. Coding gene and RNA sequences were predicted through Prodigal [23] and Barrnap (http://www.vicbioinformatics.com/software.barrnap.shtml). Functional categories were predicted through the BLASTKoala web tool using the KEGG database [26]. Genomic islands were expected through Island Viewer 4 server and represented along with the genomic map [3].

### 2.6 Data availability

The genome of *Shewanella algae* 2NE11 was deposited under the NCBI GenBank accession number CP055159. Raw data of sequencing is available in the Sequence Read Archive (SRA) repository of the National Center for Biotechnology Information (NCBI) under the accession number PRJNA547647. Complete information about strain 2NE11 is on the biosample SAMN15232066.

## 3 Results

### 3.1 Organism information

#### 3.1.1 Description of *Shewanella algae* 2NE11

The main physiological and biochemical features of *S. algae* 2NE11 were described to understand its metabolism and identify features that allows the strain to enhance its decolorization capacity. Parameters evaluated during sample collection show that it had a DBO of 13100 mg/L and a DQO of 36900 mg/L. *Shewanella algae* 2NE11 has a doubling time of 5.03h and grows in optimal conditions at pH 6-9, between 30 and 40°C, and with 0-4% of NaCl. A more detailed description and comparison with the reference strain S. *algae* ATCC 51192 is depicted in Table 1.

**Table 1:** Physiological and biochemical features of *S.algae* 2NE11 (The reference strain ATCC 51192T data was taken from different studies [19, 35].

	***S.algae* ATCC 51192^T^**	***S.algae* 2NE11**
Oxidase	+	+
Catalase	+	+
Motility	+	+
Hemolysis	+	+
Gelatinase production	+	-
Lysine decarboxylase	-	-
H_2_S production	+	+
Nitrate reduction	+	-
**Growth at:**		
4°C	-*	+
37°C	+	+
40°C	+	+
0% NaCl	-	+
6% NaCl	+	+
10% NaCl	+	+
**Utilization of:**		
Glucose	+	-
D-fructose	-	-
Maltose	-	+
L-arabinose	-	+
Citrate	+\-	-
Sucrose	-	+
N-acetyl glucosamine	⁎⁎	+
DL-lactate	⁎⁎	+
% GC	52.4	52.98

⁎ : No growth in 24h;

⁎⁎ : Not evaluated

#### 3.1.2 Decolorization of synthetic dyes

In this research, we evaluated the decolorization rate of strain 2NE11 under the exposure of various types of dyes to elucidate its potential in bioremediation.

**Figure 1:**
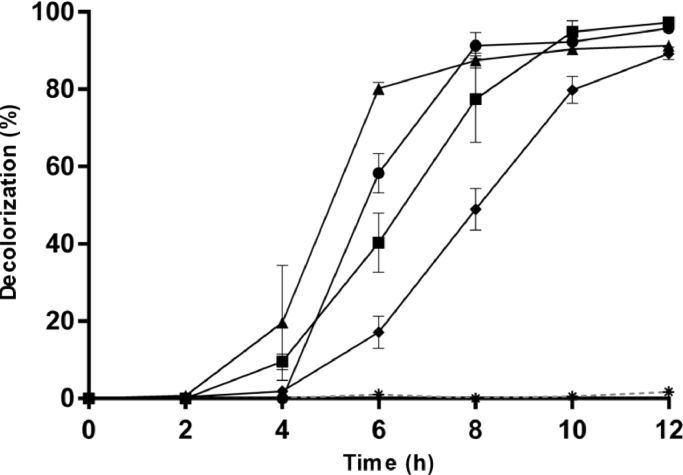
Decolorization kinetic by S. algae 2NE11. The graph shows the decolorization of Methyl Orange (●), Bright Blue Remazol (■), Yellow Procion HEXL (▲), Direct Blue 71 (♦), and control (*). Each measure in the kinetic contains a standard deviation bar.

The strain 2NE11 decolorizes azo and anthraquinone dyes like Methyl Orange (95.76%), Bright Blue Remazol (97.29%), Yellow Procion HEXL (91.31%), and Direct Blue 71 (89.24%) at 12 h (Figure 1). This strain can reduce azo dyes ranging from monoazo dyes such as Methyl Orange and triazo dyes such as Direct Blue 71. It has also been shown to be effective against anthraquinone dyes such as Bright Blue Remazol. However, the time required to decolorize may increase depending on dye complexity, as shown when comparing the decolorization kinetics of Methyl Orange and Direct Blue 71 in our results (Figure 1).

### 3.2 Genome sequencing information

#### 3.2.1 Chromosome features

**Figure 2:**
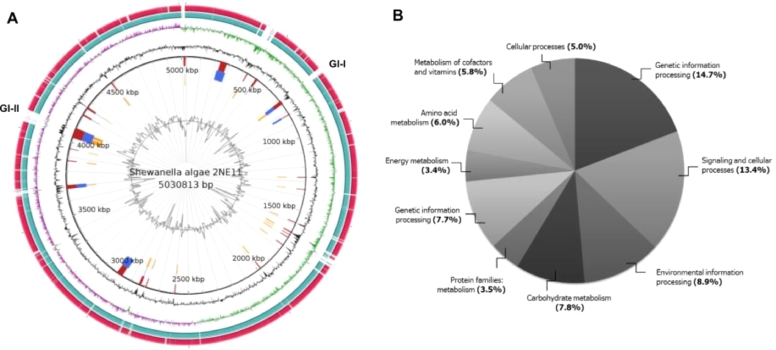
A. Visualization of S. algae 2NE11 genome compared with S. algae KC-Na-R1 (NZ_CP033575.1) and S. algae CCU101 (NZ_CP018456.1). It shows genomic island prediction in two positions (GI-I and GI-II). Starting from the inner circle moving outwards, the following tracks are shown: GC content (Black), GC skew– (Purple), GC skew+ (Green), S. algae KC-Na-R1 genome (light blue), S. algae CCU101 (ed). B. Bar plot showing the principal functional categories of strain 2NE11 according to the KEGG Orthology is depicted.

We performed whole-genome sequencing and genomic analyses to gain insights into the genomic features associated with synthetic dye decolorization. Raw data of strain 2NE11 contains 141,935 subreads with a Subread N50 of 15,524 and an average subread length of 10,574. The *Shewanella algae* 2NE11 genome was fully circularized into 5,030,813 bp with 231.29x coverage, and 52.98 %GC content. It was deposited in the GenBank database under accession number CP055159. It was not found evidence of plasmids. Genome features of *S. algae* 2NE11 and its comparison with other strains are depicted in Table 2. Functional categories analysis revealed genes related to genetic information, signaling, cellular and environmental information processing as the most representative inside the whole genome (Figure 2). Also, it is necessary to mention that it was not possible to conclude the presence of complete prophage regions inside the genome.

**Table 2:** Genome features of *Shewanella algae* 2NE11 and other strains related to.

**Feature**	**2NE11**	**ATCC 51192**	**RQs-106**	**KC-Na-R1**	**TUM4442**	**CECT 5071**	**150735**
Genome size (bp)	5,030,813	4,978,360	4,990,025	5,036,300	4,798,767	4,924,764	5,070,545
Number of contigs	1	52	1	1	1	1	1
Total genes	4,475	4,505	4,430	4,715	4,264	4,400	4,528
CDSs	4,334	4,392	4,296	4,582	4,130	4,264	4,392
Protein coding genes	4,288	4,329	4,253	4,527	4,098	4,225	4,357
tRNAs	111	95	105	102	105	107	107
rRNAs	25	14	25	25	25	25	25
ncRNAs	5	4	4	6	4	4	4
Pseudo genes	46	63	43	55	32	39	35
GenBank accession	CP055159	JAAXPX000000000	CP046378	CP033575	AP024610	CP068230	CP068229

Two genomics islands (GI-I and GI-II) were predicted and depicted in Figure 2. GI-I has a length of 25,322 bp and comprises 21 genes, whereas GI-II has a size of 70,550 bp and consists of 64 genes. GI-I contains mainly conjugative transfer proteins, and GI-II has proteins of the type IV secretion system, regulators transcriptional, conjugative transfer proteins, esterases, hydrolases, reductases, nitro reductases, and oxidoreductases. GI-II likely enhances its environmental adaptability because its relationship with many reduction processes and allow greater substrate diversity consumption during its respiration.

The results of the candidate genes search related to decolorization, metal resistance and carbohydrate metabolism are presented in Table 3.

**Table 3:** Candidate genes of *Shewanella algae* 2NE11 related to decolorization and other properties. The letters ^a^, ^b^ and ^c^ refer to a link between the words.

**Category**	**Gene name**	**Locus tag**	**Description**
Decolorization		HU689_20695	FMN-dependent NADH-azoreductase
		HU689_04585; HU689_04700; HU689_21345	NADPH-dependent oxidoreductase
		HU689_05310	Heme-dependent Dyp peroxidase
		HU689_08360 - HU689_08395	Operon Mtr
Metal Resistance	*cadA*	HU689_10830	P-type ATPase protein
	*corA^a^*&*corC^b^*	HU689_12865^a^&(HU689_16615; HU689_20255; HU689_07770)^b^	Magnesium and cobalt transport protein^ab^
	*zntB*	HU689_05170	Efflux Zn^+2^ ion transport protein
	*arsA^a^*&*arsB^b^*&*arsC^c^*	HU689_08030^a^&HU689_02860^b^&HU689_10495^c^	ATPase protein^a^&Arsenite efflux transporter protein^b^&Arsenate reductase^c^
Carbohydrate metabolism		HU689_06695^a^&HU689_06680^b^	L-lactate permease^a^&Lactate utilization protein^b^
		(HU689_06290, HU689_06255)^a^ (HU689_06275, HU689_06270)^b&^HU689_06250^c^&(HU689_06285, HU689_06280, HU689_06265, HU689_06260)^d^	Transport protein^a^&Catalysis protein^b^&Regulation protein^c^&Complementary processes protein^d^

## 4 Discussion

### 4.1 Description of *Shewanella algae* 2NE11

*Shewanella algae* were isolated in 1990 from a red algae (*Jainia* spp.), mainly characterized by its ability to tolerate and stimulate its growth in high salt concentrations of up to 12% [35, 41].

*S. algae* 2NE11 is a mobile microorganism, oxidase, and catalase-positive. It can produce hemolysis and H_2_S; but does not have gelatinase, lysine decarboxylase, and cannot reduce nitrates. This strain grows in optimal conditions at pH 6-9, with an optimum between 30 and 40°C, and with 0-4% NaCl. Like strain ATCC 51192, it can grow at 4, 37, 40°C, and up to 10% NaCl. However, unlike *S. algae* ATCC 51192, the strain 2NE11 does not require NaCl to cell growth [19, 35]. *S. algae* 2NE11 can use a greater variety of carbohydrates such as maltose, L-arabinose, sucrose, N-acetyl glucosamine, and DL-lactate to cell growth.

The physiological description of *S. algae* 2NE11 suggests that this strain could be used in future bioremediation studies of textile effluents since it tolerates high concentrations of salt and consumes a wide variety of carbohydrates that could be used in the biotechnological process optimization.

### 4.2 Decolorization of synthetic dyes

Synthetic dyes are classified based on their chemical structure in azo, anthraquinone, and triphenylmethane. Among these, azo dyes are the most used in the textile industry. They are classified in monoazo, diazo, triazo, and polyazo based on the number of azo bonds (N=N) within their chemical structure [11].

Some species inside the genus Shewanella, like *S. oneidensis, S. decolorationis, S. putrefaciens, S. xiamenensis,* and *S. algae,* were described for their ability to decolorize under different conditions [20, 27, 29, 34, 53, 55]. Previous reports indicate that *S. decolorationis* S12 has an efficiency of 99% after 15 h of exposure to Brilliant Blue Remazol dye (50 mg/L) [56]. This efficiency value is higher than the 97.29% obtained after 12 h of incubation of the strain 2NE11. The degradation of other more complex dyes such as Direct Blue 71 has also been previously evaluated in *Pseudomonas* strains, obtaining up to a little more than 70% efficiency after a broad incubation period of several days [17, 37]. However, with strain 2NE11, we can observe a better decolorization rate of more than 89% after only 12 hours of exposure. Other simple dyes such as methyl orange have been extensively investigated in different species, obtaining almost complete decolorization just as the strain under study [5, 8, 34].

### 4.3 Insights from the genome sequence

Table 2 shown clearly that strain 2NE11 has similar genome size with *S. algae* KC-Na-R1. It shares similar number of rRNAs, tRNAs and ncRNAs with strains RQs-106, KC-Na-R1, TUM4442, CECT 5071, and 150735. Only *S. algae* ATCC 51192 shown a different number likely due to its partial genome sequencing project. Other features like total genes number and CDS are concordant with the genome size showing a partial correspondence between strains.

### 4.4 Candidate genes for decolorization

Decolorization by microorganisms has been widely studied for a long time, identifying the main responsible protein of this process. The prediction of genes related to decolorization in strain 2NE11 gives us an overview of how its whole metabolic machinery works in decolorization. Its genome contains protein-coding genes previously associated with decolorization, such as an FMN-dependent NADH-azoreductase gene (*HU689_20695*), NADPH-dependent oxidoreductase genes (*HU689_04585; HU689_04700; HU689_21345*), and heme-dependent Dyp peroxidase gene (*HU689_05310*). The operon Mtr (*HU689_08360* - *HU689_08395*), an electron chain transport previously, encodes two *OmcA* genes (*HU689_08375, HU689_08380*) as other strains within the genus *Shewanella.*

*Shewanella algae* 2NE11 has several genes related to decolorization in other studies, such as the FMN-dependent protein NADH-azoreductase. *HU689_20695* gene has a length of 594bp and codify an azoreductase of 197aa that is mainly related with WP_025888486 and WP_025010143 (*Shewanella* sp.). AzoR (*WP_011074045*) of the reference specie *S. oneidensis* is the closer gene intraspecie with only 72.02% of identity, suggesting that azoreductase gene of *S. algae* could diverge in a different group of the well know AzoR*.* Azoreductase is widely spread inside the genus algae, such as strains RQs-106 (*GMX02_01695*), TUM4442 (*TUM4442_38770*), CECT 5071 (*E1N14_020345*), KC-Na-R1 (*EEY24_23150*), and 150735 (*JKK46_01670*). It has been previously described for its ability to bio-transform and detoxify azo dyes and aromatic amines by reducing azo bonds [32]. This mechanism has been related to flavin-dependent enzymes, and it is even considered that flavin could improve the enzyme thermal stability [30].

*HU689_05310* gene present a length of 936bp, and it is related with a dyp-peroxidase of 311aa. This protein is principally related with WP_025886988 (*Shewanella* sp.) and WP_101096621 (*S. indica*) with a percentage identity of 99.68% and 99.36% respectively. This type of peroxidase has been reported in previous studies as efficient degrader of mainly anthraquinone dyes. The strain under study 2NE11 would be mediating the degradation of the Brilliant Blue Remazol dye through the catalytic activity of the dyp-peroxidase (*HU689_05310*) found in its genome. However, the recent discovery of its possible multifunctional activity as a hydrolytic agent does not allow us to conclude the biochemical process through which decolorization would perform [12, 30, 44]. Likewise, other potentially enzymes such as NADPH-dependent oxidoreductases were found with 189aa (*HU689_04585*), 200aa (*HU689_04700*), 218aa (*HU689_21345*). They would be involved in FMNH_2_-dependent reduction to various metabolites related to stress response and iron reduction [45].

Other proteins that have also got much relevance within decolorization are the electron transport chain of the operon Mtr [6, 28]. That has been associated with extracellular decolorization processes and linked with polar azo dyes. The operon Mtr is composed of proteins in the inner membrane, outer membrane, and the periplasm that would allow energy conservation and electrons transport in the cell membrane. Previous reports indicate that the operon Mtr is highly diverse among *Shewanella* species due to gene duplication, acquisition, and loss. Furthermore, some of its compounds, such as the *MtrA, OmcA*, and *OmcB* genes, play an essential role in electron transport, which they are highly conserved [21, 59]. The mentioned would support the idea that *OmcA* gene duplication in the strain 2NE11, as in other *Shewanella algae* strains, could give it a greater capacity to transfer electrons and improve its decolorization ability in contrast to other species in the genus.

### 4.5 Candidate genes for metal resistance

The heavy metal resistance improves a microorganism potential in the textile effluent bioremediation because they are frequently found as a component in the textile industry wastewater. Based on genome analysis of the strain 2NE11, we found that the *cadA* gene (*HU689_10830*) encodes a P-type ATPase protein of 798 aa length, which has been described previously as a determinant that allows resistance to cadmium through a decrease in the intracellular accumulation of this cation, mediated by active transport [48, 49]. The *HU689_10830* gene is mainly related with *S. algae* TUM17384 (*AP024616*) and strain 150735 (CP068229) with an identity of 99.58 and 99.42%.

Magnesium is a cation widely used by living organisms because it allows various biological functions such as genome stability, a cofactor for ATP hydrolysis, and DNA replication [22]. We found that strain 2NE11 also contains genes related to magnesium and cobalt transport such as *corA* (*HU689_12865*) and *corC* (*HU689_16615; HU689_20255; HU689_07770*), widely associated with the exterior and interior flux of Mg^+2^ and Co^+2^ ions in gram-negative and positive bacteria [15]. Resistance to other ions such as zinc was also evaluated, allowing identify an efflux Zn^+2^ ion transport encoded by the *zntB* gene (*HU689_05170*), closely related to strain 18064-CSB-B-B (*CP047422*) and ATCC 49138 (*AP024609*) with 99.17% identity. This would allow maintenance of zinc concentration narrow under the limits necessary for living cells [52]. Previous study in *Shewanella* sp. strain ANA-3 have shown that arsenate resistance occurs via two different pathways: detoxification and arsenate respiratory reduction [33]. Nevertheless, none of them could be found completely in *S. algae* 2NE11. Only, some arsenate resistance genes such as ATPase protein *arsA* (*HU689_08030*), arsenite efflux transporter *arsB* (*HU689_02860*), and arsenate reductase *arsC* (*HU689_10495*) were found in the genome of strain 2NE11; however, their lack of operon-shaped structure prevents us from concluding that they are expressed or functional.

### 4.6 Candidate genes for carbohydrate metabolism

Carbohydrates have been widely studied in the optimization of dyes degradation process due it represents a carbon source which improves cell growth. According to genomic annotation, we assume that DL-lactate metabolism in *S. algae* 2NE11 could be associated with an L-lactate permease activity (*HU689_06695*) and a lactate utilization protein (*HU689_06680*). Additionally, we found genes that could be related to the catabolic pathway of N-acetylglucosamine, involved in processes such as transport (*HU689_06290, HU689_06255*), catalysis (*HU689_06275, HU689_06270*), regulation (*HU689_06250*), and complementary processes (*HU689_06285, HU689_06280, HU689_06265, HU689_06260*) due their highly similarity to *S. oneidensis* MR-1 Nag genes. They have been found in almost all genomes of *Shewanella* genus and many isolated strains have been able to grown in N-acetylglucosamine as sole carbon source, ability also shared by 2NE11 [38]*.* Moreover, Nag genes has been recently related to glucose metabolism of *Shewanella oneidensis* MR-1 for enhanced pollutant degradation [10]. Likewise, it has been reported in multiple investigations that lactate can be used as electron acceptor in cellular respiration, allowing to improve the dye degradation process required [7, 56].

Although the diversity of carbon source utilization in *Shewanella* is limited, the metabolic machinery for the catalysis of the other carbohydrates could not be fully elucidated in *S. algae* 2NE11. It has already been confirmed that several strains of *Shewanella* can improve the decolorization efficiency when grown under additives such as carbohydrates [10]. *S. algae* 2NE11 seems to have the complete genomic machinery necessary to metabolize lactate and N-acetylglucosamine, which has been related to the results obtained by physiological tests. Previous studies indicate that they are promoters of the decolorization efficiently, for which we highlight that these two sugars could be used to optimize the decolorization process on a larger scale with strain 2NE11 [10, 55, 58].

## 5 Conclusion

We present the complete genome sequence and physiological profile of *S. algae* 2NE11, a bacteria dye-degrading isolated from an industrial effluent in Peru. It can tolerate up to 10% NaCl, and it can decolorize azo and anthraquinone dyes. The molecular decolorization mechanisms involved include the catalytic activity of azoreductases, dyp-peroxidases, oxidoreductases, and the Mtr respiratory pathway. Likewise, we identified several genes related to metal resistance and carbohydrate metabolism, which would enhance their potential applicability in textile effluents bioremediation. This explorative view of strain 2NE11 reveals interesting genomic features, however future research is required to show functionality.

## Authors contributions

Wendy Lizárraga & Carlo Mormontoy & Ruth García & Pablo Ramírez consigned and designed the experiment. Wendy Lizárraga performed and participated in all experiments. Carlo Mormontoy performed the phylogenetic tree and participated in the analysis of the data. Mario Taira & Hedersson Calla & Maria Castañeda performed part of the physiological tests. Claudia Marin sampled and isolated the strain under study. Michel Abanto participated in the assembly of the genome. All authors participated in the final revision of the manuscript.

## Declaration of Competing Interests

The authors declare the following financial interests/personal relationships which may be considered as potential competing interests: Pablo Ramirez reports financial support was provided by Consejo Nacional de Ciencia, Tecnología e Innovación Tecnológica. Wendy Lizarraga reports financial support was provided by National University of San Marcos Biological Sciences Faculty. Pablo Ramirez reports a relationship with Consejo Nacional de Ciencia y Tecnología that includes: funding grants. Pablo Ramirez reports a relationship with National University of San Marcos Biological Sciences Faculty that includes: employment, funding grants, and speaking and lecture fees.

## Appendix Supplementary materials

Image, application 1
