# Supplementary material for: Complete genome sequence of Shewanella algae strain 2NE11, a decolorizing bacterium isolated from industrial effluent in Peru
Source: Biotechnol Rep (Amst). 2022 Jan 31;33:e00704. doi: 10.1016/j.btre.2022.e00704 (PMC8816663; doi:10.1016/j.btre.2022.e00704)
Supplement: Supplementary file 1 [file mmc1.docx]

| **Genomic Island I** | | |
| --- | --- | --- |
| **Locus_tag** | **Position (pb)** | **Product** |
| HU689_RS03215 | 716238..717204 | integrase domain-containing protein |
| HU689_RS03220 | 717516..717768 | hypothetical protein |
| HU689_RS03225 | 718001..718256 | hypothetical protein |
| HU689_RS03230 | 718334..718652 | hypothetical protein |
| HU689_RS03235 | 718753..719023 | hypothetical protein |
| HU689_RS03240 | 719063..719222 | hypothetical protein |
| HU689_RS03245 | 719488..719719 | hypothetical protein |
| HU689_RS03250 | 719715..720591 | hypothetical protein |
| HU689_RS03255 | 720800..724238 | conjugal transfer protein TraG N-terminal domain-containing protein |
| HU689_RS03260 | 724234..724591 | hypothetical protein |
| HU689_RS03265 | 724803..727155 | type I restriction enzyme HsdR N-terminal domain-containing protein |
| HU689_RS03270 | 727199..727460 | hypothetical protein |
| HU689_RS03275 | 727631..727820 | hypothetical protein |
| HU689_RS03280 | 727822..728383 | regulator |
| HU689_RS03285 | 728479..731173 | conjugal transfer mating pair stabilization protein TraN |
| HU689_RS03290 | 731629..734782 | SIR2 family protein |
| HU689_RS03295 | 735015..735612 | tyrosine-type recombinase/integrase |
| HU689_RS03300 | 736047..738207 | NTPase KAP |
| HU689_RS03305 | 738322..738790 | hypothetical protein |
| HU689_RS03310 | 739139..740267 | IS110 family transposase |
| HU689_RS03315 | 740459..741560 | site-specific integrase |

**Suplementary material**

| **Genomic Island II** | | |
| --- | --- | --- |
| **Locus_tag** | **Position (pb)** | **Product** |
| HU689_RS18005 | Not specified | DUF3293 domain-containing protein* |
| HU689_RS18010 | 4031616..4032333 | two-component system response regulator ArcA |
| HU689_RS18015 | 4032900..4033692 | hypothetical protein |
| HU689_RS18020 | 4033656..4033923 | DUF2274 domain-containing protein |
| HU689_RS18025 | 4033919..4035209 | TrbI/VirB10 family protein |
| HU689_RS18030 | 4035211..4036204 | P-type conjugative transfer protein TrbG |
| HU689_RS18035 | 4036200..4036905 | conjugal transfer protein TrbF |
| HU689_RS18040 | 4036935..4038303 | P-type conjugative transfer protein TrbL |
| HU689_RS18045 | 4038315..4039059 | P-type conjugative transfer protein TrbJ |
| HU689_RS18050 | 4039085..4041518 | conjugal transfer protein TrbE |
| HU689_RS18055 | 4041530..4041815 | VirB3 family type IV secretion system protein |
| HU689_RS18060 | 4041811..4042189 | TrbC/VirB2 family protein |
| HU689_RS18065 | 4042191..4043220 | P-type conjugative transfer ATPase TrbB |
| HU689_RS18070 | 4043216..4043693 | ribbon-helix-helix protein, CopG family |
| HU689_RS18075 | 4043689..4045699 | conjugal transfer protein TraG |
| HU689_RS18080 | 4045934..4046213 | EexN family lipoprotein |
| HU689_RS18085 | 4046209..4047154 | LysR family transcriptional regulator |
| HU689_RS18090 | 4047347..4047818 | cupin domain-containing protein |
| HU689_RS18095 | 4047849..4048803 | prolyl oligopeptidase family serine peptidase |
| HU689_RS18100 | 4048945..4050193 | aldo/keto reductase |
| HU689_RS18105 | 4050246..4050927 | nitroreductase |
| HU689_RS18110 | 4050947..4051940 | aldo/keto reductase |
| HU689_RS18115 | 4051976..4053368 | NAD-dependent succinate-semialdehyde dehydrogenase |
| HU689_RS18120 | 4053472..4054378 | SDR family NAD(P)-dependent oxidoreductase |
| HU689_RS18125 | 4054423..4055161 | SDR family oxidoreductase |
| HU689_RS18130 | 4055207..4055804 | flavodoxin |
| HU689_RS18135 | 4055820..4056810 | aldo/keto reductase |
| HU689_RS18140 | 4057068..4057971 | LysR family transcriptional regulator |
| HU689_RS18145 | 4057974..4058868 | LysR family transcriptional regulator |
| HU689_RS18150 | 4059190..4059967 | carboxymuconolactone decarboxylase family protein |
| HU689_RS18155 | 4059969..4061058 | alpha/beta fold hydrolase |
| HU689_RS18160 | 4061126..4062782 | carboxylesterase/lipase family protein |
| HU689_RS18165 | 4062876..4063977 | alpha/beta hydrolase |
| HU689_RS18170 | 4064032..4065583 | cation:proton antiporter |
| HU689_RS18175 | 4065602..4066736 | MBL fold metallo-hydrolase |
| HU689_RS18180 | 4066915..4067689 | SDR family oxidoreductase |
| HU689_RS18185 | 4067788..4068199 | cupin domain-containing protein |
| HU689_RS18190 | 4068280..4069720 | MFS transporter |
| HU689_RS18195 | 4070307..4072275 | relaxase/mobilization nuclease and DUF3363 domain-containing protein |
| HU689_RS18200 | 4072714..4073311 | S26 family signal peptidase |
| HU689_RS18205 | 4073307..4073841 | DUF2840 domain-containing protein |
| HU689_RS18210 | 4073837..4074089 | chromosome partitioning protein ParB |
| HU689_RS18215 | 4074085..4074724 | AAA family ATPase |
| HU689_RS18220 | 4075010..4075865 | replication initiator protein A |
| HU689_RS18225 | 4075891..4076176 | helix-turn-helix domain-containing protein |
| HU689_RS18230 | 4076259..4077054 | DUF2285 domain-containing protein |
| HU689_RS18235 | 4077376..4077706 | DUF2958 domain-containing protein |
| HU689_RS18240 | 4077991..4078279 | helix-turn-helix transcriptional regulator |
| HU689_RS18245 | 4078406..4079579 | SIR2 family protein |
| HU689_RS18250 | 4079575..4081378 | ATP-binding protein |
| HU689_RS18255 | 4081473..4081788 | DUF736 domain-containing protein |
| HU689_RS18260 | 4082520..4083276 | hypothetical protein |
| HU689_RS18265 | 4083636..4085661 | ParB/RepB/Spo0J family partition protein |
| HU689_RS18270 | 4085741..4086569 | DUF945 domain-containing protein |
| HU689_RS18275 | 4087168..4087978 | hypothetical protein |
| HU689_RS18280 | 4088018..4088528 | DNA repair protein RadC |
| HU689_RS18285 | 4088994..4089690 | helix-turn-helix transcriptional regulator |
| HU689_RS18290 | 4089714..4090845 | DUF1016 domain-containing protein |
| HU689_RS18295 | 4090841..4092041 | tyrosine-type recombinase/integrase |
| HU689_RS18300 | 4092264..4094355 | elongation factor G |
| HU689_RS18305 | 4094616..4094886 | hypothetical protein |
| HU689_RS18310 | 4094882..4096049 | diguanylate cyclase |
| HU689_RS18315 | Not specified | IS4 family transposase* |
| HU689_RS18320 | 4096618..4101172 | ATP-grasp domain-containing protein |

*: Island viewer not specify the initial and final position of the sequence.
